# Renewal approach for the energy–momentum relation of the Fröhlich polaron

**DOI:** 10.1007/s11005-023-01711-w

**Published:** 2023-08-18

**Authors:** Steffen Polzer

**Affiliations:** University of Geneva, Rue du Conseil-Général 7-9, 1205 Geneva, Switzerland

**Keywords:** Polaron, Energy–momentum relation, Renewal theory, Point processes, 81Q10, 81T10, 60K05, 60G55

## Abstract

We study the qualitative behaviour of the energy–momentum relation of the Fröhlich polaron at fixed coupling strength. Among other properties, we show that it is non-decreasing and that the correction to the quasi-particle energy is negative. We give a proof that the effective mass lies in $(1, \infty )$ that does not need the validity of a central limit theorem for the path measure.

## Introduction and results

The polaron models the interaction of an electron with a polar crystal. The Fröhlich Hamiltonian describing the interaction of the electron with the lattice vibrations has a fibre decomposition in terms of the Hamiltonians

$$\begin{aligned} H(P) = \frac{1}{2} (P - P_f)^2 + {\textbf{N}} + \frac{\sqrt{\alpha }}{\sqrt{2} \pi } \int _{{\mathbb {R}}^3} \frac{1}{|k|}(a_k + a^*_k) \, {\textrm{d}}k \end{aligned}$$

at fixed total momentum $P\in {\mathbb {R}}^3$ that act on the bosonic Fock space over $L^2({\mathbb {R}}^3)$. Here $a_k^*$ and $a_k$ are the creation and annihilation operators satisfying the canonical commutation relations $[a^*_k, a_{k'}] = \delta (k-k')$, ${\textbf{N}} \equiv \int _{{\mathbb {R}}^3} a^*_k a_k\, {\textrm{d}} k$ is the number operator, $P_f \equiv \int _{{\mathbb {R}}^3} k a_k^* a_k {\textrm{d}}k$ is the momentum operator of the field and $\alpha >0$ is the coupling constant. Of particular interest has been the energy–momentum relation

$$\begin{aligned} E(P):= \inf {\text {spec}}(H(P)). \end{aligned}$$

For small |*P*| the system is believed to behave like a free particle with an increased “effective mass”. *E* is known to have a strict local minimum at $P=0$ and to be smooth in a neighbourhood of the origin. The effective mass is defined as the inverse of the curvature at the origin such that

1 $$\begin{aligned} E(P) - E(0) = \frac{1}{2m_{\text {eff}}} |P|^2 + o(|P|^2) \end{aligned}$$

in the limit $P \rightarrow 0$. While significant effort has been put into the study of the asymptotic behaviour of *E*(0) and $m_{\text {eff}}$ in the strong coupling limit $\alpha \rightarrow \infty$ (see e.g. [3, 5, 7, 13–15, 22]), we will be interested in the qualitative behaviour of *E* at a fixed value of the coupling constant. One valuable tool for the analysis of *E*(0) and $m_{\text {eff}}$ has been their probabilistic representation obtained via the Feynman–Kac formula. The approach taken below extends the probabilistic methods developed for the analysis of the effective mass to the whole energy–momentum relation. Let

$$\begin{aligned} E_\text {ess}(P):= \inf \text {ess spec}(H(P)) \end{aligned}$$

be the bottom of the essential spectrum. It is known [24] that

$$\begin{aligned} E_\text {ess}(P) = E(0) + 1 \end{aligned}$$

for all *P*. From now on, we will often abuse notation and identify a radially symmetric function on ${\mathbb {R}}^3$ with a function on $[0, \infty )$. Keeping that in mind, let

$$\begin{aligned} {\mathcal {I}}_0:= \{P\in [0, \infty ): E(P) < E(0) + 1\} \end{aligned}$$

(which is known to contain a neighbourhood of the origin). For Hamiltonians with stronger regularity assumptions (e.g. the Fröhlich polaron with an ultraviolet cutoff), it is known [17] that the spectral gap closes in the limit, i.e. that $\lim _{P \rightarrow \infty } E(P) = E_\text {ess}(0)$. For the Fröhlich polaron in dimensions 1 and 2 it is known [24] that ${\mathcal {I}}_0 = [0, \infty )$, i.e. that the spectral gap does not close in a finite interval. In dimension 3, however, it has been predicted in the physics literature that ${\mathcal {I}}_0$ is bounded [9]. For sufficiently small coupling constants, this has been shown in [6]. In the framework presented below, the question whether ${\mathcal {I}}_0$ is bounded or unbounded reduces to the study of the tails of a probability distribution on $(0, \infty )^2$. There does not seem to be known much about the behaviour of *E* in the intermediate *P*-regime. In [8] it was shown that *E* is real analytic on ${\mathcal {I}}_0$ with $E(0) \le E(P)$ for all *P* and that the inequality is strict for *P* outside of a compact set. In recent work, it has been shown [11] that *E* has indeed a strict global minimum in 0. In the present text, we will prove some previously unknown properties of *E*, namely monotonicity and concavity of $P\mapsto E(\sqrt{P})$ on $[0, \infty )$, both of which are strict on ${\mathcal {I}}_0$. The (strict) monotonicity additionally allows us to replicate the result of [11]. We denote by ${\text {cl}}({\mathcal {I}}_0)$ the closure of ${\mathcal {I}}_0$.

### Theorem 1

The following holds.

(i) $P \mapsto E(P)$ is non-decreasing on $[0, \infty )$ and strictly increasing on ${\mathcal {I}}_0$. In particular, ${\mathcal {I}}_0$ is an (potentially unbounded) interval.
(ii) $P \mapsto E(\sqrt{P})$ is strictly concave on ${\mathcal {I}}_0$. In particular,
  $$\begin{aligned} E(P) - E(0) < \frac{1}{2m_{\text {eff}}} P^2 \end{aligned}$$
  for all $P>0$, i.e. the correction to the quasi-particle energy is negative and $\big [0, \sqrt{2 m_\text {eff}}\, \big ) \subset {\mathcal {I}}_0$.
(iii) For $|P| \notin {\text {cl}}({\mathcal {I}}_0)$ we have $\lim _{\lambda \uparrow E(P)}\langle \Omega , (H(P) - \lambda )^{-1} \Omega \rangle <\infty$, where $\Omega$ is the Fock vacuum and in particular *H*(*P*) does not have a ground state.

For the polaron with an ultraviolet cut-off and in dimensions 3 and 4, the non-existence of a ground state for $|P|\notin {\mathcal {I}}_0$ has been shown in [17]. In a certain limit of strong coupling, the negativity of the correction to the quasi-particle energy has been shown in [15]. In (iii), we used that if *H*(*P*) has a ground state, then it is non-orthogonal to $\Omega$: The operator $e^{{\textrm{i}} \pi {\textbf{N}}} e^{-TH(P)} e^{-{\textrm{i}} \pi {\textbf{N}}}$ is for all $T>0$ positivity improving [16, Theorem 6.3] which in turn implies that if there exists a ground state $\psi _P$ of *H*(*P*), then it is unique (up to a phase) and can be chosen such that $e^{{\textrm{i}} \pi {\textbf{N}}}\psi _P$ is strictly positive [16, Theorem 2.12]. In [16, Theorem 6.4], it was shown that there exists a ground state of *H*(*P*) for $|P|< \sqrt{2}$. Part (ii) of our Theorem 1 allows us to improve this to existence of a ground state for $|P|< \sqrt{2m_\text {eff}}$.

Before starting with the proof of Theorem 1, we give a brief summary of our approach. An application of the Feynman–Kac formula to the semigroup generated by the Hamiltonian yields [8]

$$\begin{aligned} \langle \Omega , e^{-TH(P)} \Omega \rangle = \int _{C([0, \infty ), {\mathbb {R}}^3)} {\mathcal {W}}({\textrm{d}}X)\, e^{- {\textrm{i}} P \cdot X_{0,T}} \exp \bigg ( \frac{\alpha }{2} \int _0^T \int _0^T {\textrm{d}}s {\textrm{d}}t \, \frac{e^{-|t-s|}}{|X_{s, t}|} \bigg ) \end{aligned}$$

for all $P\in {\mathbb {R}}^3$ and $T\ge 0$, where $\Omega$ is the Fock vacuum, ${\mathcal {W}}$ is the distribution of a three-dimensional Brownian motion started in the origin and $X_{s, t}:= X_t -X_s$ for $X\in C([0, \infty ), {\mathbb {R}}^3)$ and $s, t\ge 0$. After normalizing the expression above by dividing by $\langle \Omega , e^{-TH(0)} \Omega \rangle$, one can study *E* by looking at the large *T* asymptotics of Brownian motion perturbed by a pair potential. Herbert Spohn conjectured in [23] convergence of the resulting path measure under diffusive rescaling to Brownian motion and showed that the respective diffusion constant is then the inverse of the effective mass, see also [8]. The validity of this central limit theorem was shown in [2, 19, 20] by using the point process representation of the path measure that has been introduced by Mukherjee and Varadhan [20]. There the path measure is represented as a mixture of Gaussian measures, where the mixing measure can be expressed in terms of a perturbed birth and death process. An application of renewal theory then yielded the existence of an infinite volume measure and a central limit theorem provided that a certain technical condition holds. The validity of said condition was first verified for sufficiently small $\alpha$ in [20] and then for all $\alpha$ in [2] (where known spectral properties of *H*(0) are used) and independently in [19] (where a purely probabilistic proof is given). The proof given in [2] uses the point process representation in order to derive a renewal equation for $T \mapsto \langle \Omega , e^{-TH(0)} \Omega \rangle$. The condition of Mukherjee and Varadhan was then shown to be equivalent to the known existence of a ground state of *H*(0) that is non-orthogonal to $\Omega$. We will use a similar approach and derive renewal equations for $T \mapsto \langle \Omega , e^{-TH(P)} \Omega \rangle$ for any *P*. We arrive at our results by comparing the asymptotic behaviour of the solutions in dependency of *P*.

In our units, the free electron has mass 1 and physically one would expect that $1< m_{\text {eff}} < \infty$. The proof of the central limit theorem entails a formula for the diffusion constant that directly implies that this indeed holds. We will give an additional proof that yields (essentially) the same formula for the effective mass but that does not rely on the validity of a central limit theorem. Numerous efforts have been made to establish central limit theorems for related models (see e.g. [2, 4, 10, 18]) and a generalization of the method presented below may be a viable alternative to study the effective mass with probabilistic methods.

## Proof of Theorem 1

We define $\triangle := \{(s, t)\in \mathbb [0, \infty )^2:\, s<t\}$ and ${\mathcal {Y}}:= \bigcup _{n=0}^\infty (\triangle \times [0, \infty ))^n$, and equip the latter with the disjoint-union $\sigma$-algebra (i.e. the final $\sigma$-algebra with respect to the canonical injections $(\triangle \times [0, \infty ))^n \hookrightarrow {\mathcal {Y}}$, $n\in {\mathbb {N}}$). For $\zeta = ((s_i, t_i, u_i))_{1\le i \le n}\in {\mathcal {Y}}$ let

$$\begin{aligned} T_1(\zeta ):= \sup _i t_i, \quad \sigma ^2(\zeta ):= {\text {dist}}_{L^2}\Big (B_{T_1(\zeta )}, {\text {span}}\{u_i B_{s_i, t_i} + Z_i: \, 1\le i \le n\}\Big )^2 \end{aligned}$$

where $(B_t)_{t\ge 0}$ is a one-dimensional Brownian motion and $(Z_n)_n$ is an iid sequence of centred Gaussian random variables with variance 1 that is independent of $(B_t)_{t\ge 0}$. For a measure $\mu$ on ${\mathcal {Y}}$ and a measurable function $f:{\mathcal {Y}} \rightarrow {\mathbb {R}}$ we abbreviate $\mu (f):= \int _{{\mathcal {Y}}} \mu ({\textrm{d}}\zeta ) f(\zeta )$ provided that the integral exists. Additionally, we set $f_P(T):= \langle \Omega , e^{-TH(P)} \Omega \rangle$ for $P\in {\mathbb {R}}^3$, $T\ge 0$.

### Proposition 2

There exists a measure $\mu$ on ${\mathcal {Y}}$ such that

$$\begin{aligned} f_P(T) = \mu \big (e^{-P^2 \sigma ^2/2} f_P(T-T_1)\mathbbm {1}_{\{T_1 \le T\}}\big ) + e^{-P^2T/2} \end{aligned}$$

holds for all $P \in {\mathbb {R}}^3$ and $T \ge 0$.

### Proof

We define

$$\begin{aligned} F_P(T_1, T_2, \xi ):= \int {\mathcal {W}}({\textrm{d}} X) \, e^{- {\textrm{i}} P \cdot X_{T_1, T_2}} \prod _{i=1}^{n} |X_{s_i, t_i}|^{-1} \end{aligned}$$

for $T_1, T_2 \ge 0$ and $\xi = ((s_i,t_i))_{1\le i \le n}$ such that the integral is well defined. Let $\nu _T({\textrm{d}}s {\textrm{d}}t):= \alpha e^{-|t-s|} \mathbbm {1}_{\{0<s<t<T\}} {\textrm{d}}s {\textrm{d}}t$. Expanding the exponential into a series and exchanging the order of integration leads to^1^

2 $$\begin{aligned} f_P(T)&= \int {\mathcal {W}}({\textrm{d}} X) \, e^{-{\textrm{i}} P \cdot X_{0, T}} \exp \left( \int \nu _T({\textrm{d}}s {\textrm{d}}t) |X_{s, t}|^{-1} \right) \nonumber \\ \nonumber&= \sum _{n=0}^\infty \frac{1}{n!} \int \nu _{T}^{\otimes n}({\textrm{d}} s_1 {\textrm{d}} t_1, \hdots , {\textrm{d}} s_n {\textrm{d}} t_n) \int {\mathcal {W}}({\textrm{d}}X)\, e^{-{\textrm{i}} P \cdot X_{0, T}} \prod _{i=1}^{n}|X_{s_i, t_i}|^{-1} \nonumber \\&= e^{c_{T}} \int \Gamma _{T}({\textrm{d}}\xi ) F_P(0, T, \xi ) \end{aligned}$$

where $\Gamma _T$ is the distribution of a Poisson point process on ${\mathbb {R}}^2$ with intensity measure $\nu _T$ and $c_T:= \nu _T({\mathbb {R}}^2)$. Let $\Gamma$ be the distribution of a Poisson point process on ${\mathbb {R}}^2$ with intensity measure $\nu ({\textrm{d}}s {\textrm{d}}t):= \alpha e^{-|t-s|} \mathbbm {1}_{\{0<s<t\}} {\textrm{d}}s {\textrm{d}}t$. By the marking theorem for Poisson point processes (see e.g. [12, Theorem 5.6]), the measure $\Gamma$ can be seen as the distribution of a birth and death process with birth rate $\alpha$ and death rate 1 (started with no individual alive at time 0) by identifying an individual that is born at *s* and that dies at *t* with the point (*s*, *t*). For $t \ge 0$ and a configuration $\xi = ((s_i, t_i))_{i}$ of individuals let $N_t(\xi ):= |\{i: \, s_i \le t < t_i\}|$ be the number of individuals alive at time *t*. By the restriction theorem for Poisson point processes (see e.g. [12, Theorem 5.2]), $\Gamma _{T}$ can be obtained by restricting $\Gamma$ to the process of all individuals that are born before *T* conditional on the event that no individual is alive at time *T*. One can easily verify that

$$\begin{aligned} e^{c_T} = e^{\alpha T} e^{-\nu ([0, T] \times (T, \infty ))} = e^{\alpha T} \Gamma (N_T = 0). \end{aligned}$$

Hence, if we denote by $$\xi _{t_1, t_2}$$ the restriction of $$\xi $$ to all individuals born in $$[t_1, t_2)$$, we can rewrite

$$\begin{aligned} f_P(T) = e^{\alpha T} \int \Gamma ({\textrm{d}}\xi ) F_P(0, T, \xi _{0, T}) \mathbbm {1}_{\{N_T(\xi ) = 0\}}. \end{aligned}$$

Let

$$\begin{aligned} \tau (\xi ):= \inf \left\{ t\ge \inf _i s_i: \, N_t(\xi ) = 0\right\} \end{aligned}$$

be the first time after the first birth at which no individual is alive. By independence of Wiener increments

$$\begin{aligned} F_P(0, T, \xi _{0, T}) = F_P(0, \tau (\xi ), \xi _{0, \tau (\xi )}) F_P(\tau (\xi ), T, \xi _{\tau (\xi ), T}), \end{aligned}$$

for all $\xi \in \{\tau \le T\}$ such that the left hand side is well defined. Let $\Xi$ be the distribution of $\xi \mapsto \xi _{0, \tau (\xi )}$ under $\Gamma$. The process $\Gamma$ regenerates after $\tau$ and by the translation invariance of $F_P$ under a simultaneous time shift in all variables and since $e^{\alpha T} = e^{\alpha \tau }e^{\alpha (T-\tau )}$

$$\begin{aligned} f_P(T) =&\int \Xi ({\textrm{d}} \xi ) \mathbbm {1}_{\{\tau (\xi ) \le T\}}e^{\alpha \tau (\xi )} F_P(0, \tau (\xi ), \xi ) f_P(T-\tau (\xi )) \\&+ e^{\alpha T}\int \Xi ({\textrm{d}} \xi ) \mathbbm {1}_{\{\tau (\xi ) > T, \, N_T(\xi ) = 0\}} F_P(0, T, \xi _{0, T}). \end{aligned}$$

The event $\{\tau > T, \, N_T = 0\}$ happens if and only if there is no birth until time *T*. Then, $\xi _{0, T}$ is the empty configuration and hence

$$\begin{aligned} F_P(0, T, \xi _{0, T}) = {\mathbb {E}}_{\mathcal {W}}\big [e^{-{\textrm{i}} P \cdot X_T}\big ] = e^{-P^2 T/2} \end{aligned}$$

for $\xi \in \{\tau > T, \, N_T = 0\}$. Under $\Xi$, the time until the first birth is ${\text {Exp}}(\alpha )$ distributed and hence $\Xi (\tau > T, \, N_T = 0) = e^{-\alpha T}$. Combined, this gives us

$$\begin{aligned} f_P(T) = e^{-P^2T/2} + \int \Xi ({\textrm{d}} \xi ) e^{\alpha \tau (\xi )} \mathbbm {1}_{\{\tau (\xi ) \le T\}} F_P(0, \tau (\xi ), \xi ) f_P(T-\tau (\xi )). \end{aligned}$$

For $(\xi , u) \in \triangle ^n \times [0, \infty )^n$, we define ${\mathbb {P}}_{\xi , u}$ by

$$\begin{aligned} {\mathbb {P}}_{\xi , u}({\textrm{d}}X):= \frac{1}{\phi (\xi , u)} e^{-\sum _{i=1}^n u_i^2 |X_{s_i, t_i}|^2/2} {\mathcal {W}}({\textrm{d}}X) \end{aligned}$$

where $\phi (\xi , u)$ is a normalization constant. Then, ${\mathbb {P}}_{\xi , u}$ is a centred and rotationally symmetric Gaussian measure and

$$\begin{aligned} \frac{1}{3}{\mathbb {E}}_{{\mathbb {P}}_{\xi , u}}\big [|X_{0,t}|^2\big ] = {\text {dist}}_{L^2}\Big (B_{t}, {\text {span}}\{u_i B_{s_i, t_i} + Z_i: \, 1\le i \le n\}\Big )^2 =: \sigma ^2_t(\xi , u) \end{aligned}$$

for all $t\ge 0$, see the proof of Proposition 3.2 in [3]. We thus have

$$\begin{aligned} F_P(0, t, \xi )&= \int {\mathcal {W}}({\textrm{d}}X) \int _{[0, \infty )^n} {\textrm{d}}u\, (2/\pi )^{n/2}\, e^{-{\textrm{i}} P \cdot X_{0,t}} e^{-\sum _{i=1}^n u_i^2|X_{s_i, t_i}|^2/2} \\&= \int _{[0, \infty )^n} {\textrm{d}}u\, (2/\pi )^{n/2} \phi (\xi , u) e^{-P^2 \sigma _t^2(\xi , u)/2}. \end{aligned}$$

Hence, the measure we are looking for is given by

3 $$\begin{aligned} \mu ({\textrm{d}} \xi {\textrm{d}}u):= \Xi ({\textrm{d}} \xi ) {\textrm{d}}u \, (2/\pi )^{n(\xi )/2} e^{\alpha \tau (\xi )} \phi (\xi , u) \end{aligned}$$

under the identification of $\triangle ^n \times [0, \infty )^n$ with $(\triangle \times [0, \infty ))^n$. $\square$

### Proposition 3

We have $\mu (e^{-\sigma ^2 P^2 /2 + E(P) T_1}) \le 1$ for all $P\in {\mathbb {R}}^3$ and for $\lambda < E(P)$ we have

$$\begin{aligned} \langle \Omega , (H(P) - \lambda )^{-1} \Omega \rangle = \frac{1}{P^2/2-\lambda } \cdot \frac{1}{1-\mu (e^{-P^2\sigma ^2 /2 + \lambda T_1})}. \end{aligned}$$

If $|P|\in {\mathcal {I}}_0$ then *E*(*P*) is the unique real number satisfying

$$\begin{aligned} \mu (e^{-P^2\sigma ^2 /2 + E(P) T_1}) = 1. \end{aligned}$$

### Proof

For $P\in {\mathbb {R}}^3$, let $\nu _P$ be the image measure of $e^{-P^2\sigma ^2(\zeta )/2}\mu ({\textrm{d}} \zeta )$ under the map $T_1$ and let $z_P(T):= e^{-P^2T/2}$ for $T \ge 0$. By Proposition 2, for any $P \ge 0$ the renewal equation

4 $$\begin{aligned} f_P = \nu _P*f_P + z_P \end{aligned}$$

holds, where the convolution $\nu _P*f_P$ is defined as

$$\begin{aligned} (\nu _P*f_P)(T):= \int _{[0, T]} \nu _P({\textrm{d}}t) f_P(T-t) \end{aligned}$$

for $T\ge 0$. As $f_P$ is continuous and strictly positive, $\inf _{0 \le t \le T} f_P(t) >0$ and hence the measure $\nu _P$ is locally finite. Renewal theory implies that the unique locally bounded solution to (4) is given by

$$\begin{aligned} f_P = \sum _{n=0}^\infty \nu _P^{*n}*z_P, \end{aligned}$$

(see e.g. [1, Theorem 2.4]). Taking the Laplace transform leads to

$$\begin{aligned} \langle \Omega , (H(P) - \lambda )^{-1} \Omega \rangle = {\mathcal {L}}(f_P)(-\lambda ) = \frac{1}{P^2/2-\lambda } \sum _{n=0}^\infty {\mathcal {L}}(\nu _P)^n(-\lambda ) \end{aligned}$$

for^2^$\lambda < E(P)$. In particular, ${\mathcal {L}}(\nu _P)(-\lambda )<1$ for $\lambda < E(P)$ and

$$\begin{aligned} \langle \Omega , (H(P) - \lambda )^{-1} \Omega \rangle = \frac{1}{P^2/2-\lambda } \cdot \frac{1}{1-\mu (e^{-P^2 \sigma ^2/2 + \lambda T_1})}. \end{aligned}$$

As mentioned earlier, if there exists a ground state of *H*(*P*) then it is unique and non-orthogonal to $\Omega$. In combination with the spectral theorem, this implies for $|P|\in {\mathcal {I}}_0$ that

$$\begin{aligned} \lim _{\lambda \uparrow E(P)} \langle \Omega , (H(P) - \lambda )^{-1} \Omega \rangle = \infty \end{aligned}$$

and thus $\mu (e^{-\sigma ^2 P^2 /2 + E(P) T_1}) = 1$ by the monotone convergence theorem. $\square$

### Remark 4

Let $P\in {\mathbb {R}}^3$ such that $|P|\in {\mathcal {I}}_0$ and let $\psi _P$ be the unique ground state of *H*(*P*). Then, by the spectral theorem,

$$\begin{aligned} \lim _{T \rightarrow \infty } f_P(T)e^{TE(P)} = \lim _{T \rightarrow \infty } \langle \Omega , e^{-T(H(P)-E(P))} \Omega \rangle = |\langle \Omega , \psi _P \rangle |^2. \end{aligned}$$

On the other hand, we can calculate this limit by using renewal theory. The key renewal theorem (see e.g. [1, Theorem 4.3]) states that for a non-lattice probability measure $\nu$ on $(0, \infty )$ and a directly Riemann integrable (e.g. Lebesgue integrable, non-negative and non-increasing) function $z:[0, \infty ) \rightarrow {\mathbb {R}}$ the unique locally bounded solution $f:[0, \infty ) \rightarrow {\mathbb {R}}$ of the renewal equation $f = \nu * f + z$ satisfies^3^

$$\begin{aligned} \lim _{T\rightarrow \infty } f(T) = \frac{\int _0^\infty z(t) \, {\textrm{d}}t}{\int _0^\infty t \, \nu ({\textrm{d}}t)}. \end{aligned}$$

Let $z_P$ and $\nu _P$ are defined as in the proof of Proposition 3 and set

$$\begin{aligned} {\hat{f}}_P(t)&:= e^{E(P)t} f_P(t) = \langle \Omega , e^{-t(H(P) - E(P))} \Omega \rangle , \\ {\hat{z}}_P(t)&:= e^{E(P)t} z_P(t) = e^{-(P^2/2- E(P))t}, \\ {\hat{\nu }}_P({\textrm{d}}t)&:= e^{E(P)t} \nu _P({\textrm{d}}t). \end{aligned}$$

Then,

$$\begin{aligned} e^{E(P)t} \cdot (\nu _p*f_P)(t) = ({\hat{\nu }}_p*{\hat{f}}_P)(t) \end{aligned}$$

for all $t\ge 0$ and hence (4) gives

$$\begin{aligned} {\hat{f}}_P = {\hat{f}}_P * {\hat{\nu }}_P + {\hat{z}}_P. \end{aligned}$$

The key renewal theorem yields

5 $$\begin{aligned} |\langle \Omega , \psi _P \rangle |^2 = \lim _{T \rightarrow \infty } {\hat{f}}_P(T) = \frac{1}{P^2/2 - E(P)} \frac{1}{\mu (T_1 e^{-P^2\sigma ^2/2 + E(P)T_1})}. \end{aligned}$$

### Corollary 5

*E* is non-decreasing and strictly increasing on ${\mathcal {I}}_0$. For $|P| \notin {\text {cl}}({\mathcal {I}}_0)$ we have $\lim _{\lambda \uparrow E(P)}\langle \Omega , (H(P) - \lambda )^{-1} \Omega \rangle <\infty$ and *H*(*P*) does not have a ground state.

### Proof

The strict monotonicity on ${\mathcal {I}}_0$ follows directly from Proposition 3. For $P_1, P_2 \notin {\mathcal {I}}_0$ with $P_1 < P_2$, we always have

$$\begin{aligned} \mu (e^{-P_2^2 \sigma ^2/2 + E_{{\text {ess}}}(0)T_1}) < \mu ( e^{-P_1^2\sigma ^2 /2 + E_{{\text {ess}}}(0)T_1}) \le 1 \end{aligned}$$

and hence

$$\begin{aligned} \mu (e^{-P^2\sigma ^2/2 + E_{{\text {ess}}}(0)T_1}) < 1 \end{aligned}$$

for all $P\in {\mathbb {R}}^3$ such that $|P| \notin {\text {cl}}({\mathcal {I}}_0)$. Hence, for those *P*

$$\begin{aligned} \lim _{\lambda \uparrow E(P)} \langle \Omega , (H(P) - \lambda )^{-1} \Omega \rangle = \frac{1}{P^2/2-E_{{\text {ess}}}(0)} \cdot \frac{1}{1-\mu (e^{-P^2 \sigma ^2 /2 + E_{{\text {ess}}}(0) T_1})} \end{aligned}$$

and *H*(*P*) does not have a ground state (since it would need to be non-orthogonal to $\Omega$). If *E* would be not non-decreasing, then ${\mathcal {I}}_0$ would not be an interval, i.e. there would exist $P_1 \in [0, \infty ) {\setminus } {\mathcal {I}}_0$ and $P_2 \in {\mathcal {I}}_0$ such that $P_1 < P_2$. This, however, would imply

$$\begin{aligned} 1 = \mu (e^{-P_2^2\sigma ^2/2 + E(P_2)T_1}) < \mu (e^{-P_1^2\sigma ^2/2 + E_\text {ess}(0)T_1}) \le 1. \end{aligned}$$

$$\square $$

### Corollary 6

The interval ${\mathcal {I}}_0$ is bounded if and only if there exists a $P\ge 0$ such that

$$\begin{aligned} \mu (e^{-P^2 \sigma ^2/2 + E_\textrm{ess}(0)T_1}) = {\widehat{\mu }}(e^{-P^2 \sigma ^2/2 + T_1}) < \infty \end{aligned}$$

where ${\widehat{\mu }}$ is the probability measure defined by ${\widehat{\mu }}({\textrm{d}} \zeta ):= e^{E(0) T_1(\zeta )} \mu ({\textrm{d}} \zeta )$.

### Proof

This easily follows from the monotone convergence theorem. $\square$

### Corollary 7

We have

6 $$\begin{aligned} m_{\textrm{eff}} = \frac{{{\widehat{\mu }}}(T_1)}{{{\widehat{\mu }}}(\sigma ^2)} \in (1, \infty ). \end{aligned}$$

### Proof

Let $P\in {\mathcal {I}}_0$ and $\lambda < E(P)$. Then,

$$\begin{aligned} \mu (e^{-P^2\sigma ^2/2 + \lambda T_1}) = 1 - \frac{1}{P^2/2 - \lambda }\cdot \frac{1}{\langle \Omega , (H(P) - \lambda )^{-1} \Omega \rangle }. \end{aligned}$$

The function $\lambda \mapsto \langle \Omega , (H(P) - \lambda )^{-1} \Omega \rangle ^{-1}$ has a removable singularity in *E*(*P*) since *E*(*P*) is for $P \in {\mathcal {I}}_0$ an isolated eigenvalue.^4^ This implies that there exists an ${{\tilde{\varepsilon }}}>0$ such that $\mu (e^{-P^2\sigma ^2/2 + (E(P) + {{\tilde{\varepsilon }}}) T_1})<\infty$. Since $\sigma ^2 \le T_1$ there thus exist $\varepsilon , \delta >0$ such that

$$\begin{aligned} \mu (e^{-(P-\delta )^2\sigma ^2/2 + (E(P) + \varepsilon )T_1}) < \infty . \end{aligned}$$

Hence, we may differentiate under the integral. Differentiating

$$\begin{aligned} 1 = \mu (e^{- P^2\sigma ^2/2 + E(P) T_1}) \end{aligned}$$

twice with respect to *P* and evaluating at $P=0$ yields the equality in (6). Notice that both integrals are finite by the previous considerations (or by (5) for that matter). Since $\sigma ^2 \le T_1$ and $\mu (\sigma ^2 < T_1)>0$, the quotient is strictly larger than 1. $\square$

### Corollary 8

$P \mapsto E(\sqrt{P})$ is strictly concave on ${\mathcal {I}}_0$. In particular,

$$\begin{aligned} E(P) - E(0) < \frac{1}{2m_{\textrm{eff}}} P^2 \end{aligned}$$

for all $P>0$, i.e. the correction to the quasi-particle energy is negative and $\big [0, \sqrt{2 m_\textrm{eff}}\, \big ) \subset {\mathcal {I}}_0$.

### Proof

For $\lambda \in \tilde{{\mathcal {I}}_0}:= \{P^2:\, P \in {\mathcal {I}}_0\}$ let $h(\lambda )$ be the unique solution to

$$\begin{aligned} \mu (e^{-\lambda \sigma ^2/2 + h(\lambda ) T_1}) = 1, \end{aligned}$$

i.e. $h = E \circ \sqrt{\cdot }$. Then, for $\lambda _1, \lambda _2 \in \tilde{{\mathcal {I}}_0}$ with $\lambda _1 \ne \lambda _2$ and $\beta \in (0, 1)$ we get with Hölders inequality with dual exponents $1/\beta$ and $1/(1-\beta )$

$$\begin{aligned}&\mu (e^{-(\beta \lambda _1 + (1-\beta )\lambda _2) \sigma ^2/2 + (\beta h(\lambda _1) + (1-\beta )h(\lambda _2)) T_1}) \\&\quad < \mu (e^{-\lambda _1 \sigma ^2/2 + h(\lambda _1) T_1})^{\beta } \mu (e^{- \lambda _2 \sigma ^2/2 + h(\lambda _2) T_1})^{1-\beta } = 1 \end{aligned}$$

which means $h(\beta \lambda _1 + (1-\beta ) \lambda _2) > \beta h(\lambda _1) + (1-\beta )h(\lambda _2)$. Hence, *h* is strictly concave on ${\mathcal {I}}_0$, which implies for all $P \in {\mathcal {I}}_0 {\setminus } \{0\}$

$$\begin{aligned} E(P) - E(0) = h(P^2) - h(0) < h^\prime (0) P^2 = \frac{1}{2}E^{\prime \prime }(0)P^2 \end{aligned}$$

where we used in the last equality that $E'(0) = 0$. $\square$

## Data Availability

Data sharing is not applicable to this article as no datasets were generated or analysed during the current study.

## Footnotes

1 Using that the integral is finite for $P=0$ shows that this is indeed justified.

2 The inequality $E(P)<P^2/2$ follows from the considerations above and can also be obtained directly from the definition of the Hamiltonians by using $E(0)<0$ and the estimate $E(P) \le \langle \psi _0, H(P) \psi _0 \rangle$, where $\psi _0$ is the ground state of *H*(0).

3 where the right hand side is by definition zero in case of an infinite denominator.

4 Then *E*(*P*) is contained in the discrete spectrum which coincides (for self-adjoint operators) with the set isolated eigenvalues of finite multiplicity [21, Theorem VII.10].
